# Unique Approach to Dental Management of Children with Hearing Impairment

**DOI:** 10.5005/jp-journals-10005-1417

**Published:** 2017-02-27

**Authors:** Navanith Renahan, R Balagopal Varma, Parvathy Kumaran, Arun M Xavier

**Affiliations:** 1 Postgraduate Trainee, Department of Pediatric Dentistry, Amrita School of Dentistry Amrita University, Kochi, Kerala, India; 2 Professor and Head, Department of Pediatric and Preventive Dentistry, Amrita School of Dentistry, Amrita University, Kochi, Kerala, India; 3 Reader, Department of Pediatric and Preventive Dentistry, Amrita School of Dentistry, Amrita University, Kochi, Kerala, India; 4 Reader, Department of Pediatric and Preventive Dentistry, Amrita School of Dentistry, Amrita University, Kochi, Kerala, India

**Keywords:** Behavior management, Communication, Deaf child, Sign language.

## Abstract

The number of deaf children has dramatically increased in the past few decades. These children present to the pediatric dentist a unique set of challenges mostly pertaining to the establishment of communication with them. There have been very few attempts in the past to break down these challenges and formulate a strategy on how to manage them effectively. This is a case report of a child who was successfully managed using two different modes of communication. Finally, the advantages and disadvantages are mentioned, and a common strategy incorporating the positives of both the methods has been devised.

**How to cite this article:**

Renahan N, Varma RB, Kumaran P, Xavier AM. Unique Approach to Dental Management of Children with Hearing Impairment. Int J Clin Pediatr Dent 2017;10(1):107-110.

## INTRODUCTION

When a deaf child walks into the dental clinic, the normal management protocol will not be appropriate. Literature does not provide specific management strategy for such children. A total of 63 million people in India have hearing impairment, which is a common cause of disability.^1^ There is no structured program to impart knowledge on how to manage such children.^2^ Sign language and treatment under general anesthesia are the routinely employed management methods. In the United Kingdom, there was an incidence of 1/1,000 births of deaf in the year 2000.^3^ Derelioglu and Yilmaz^4^ suggest that deaf children will have to be treated under general anesthesia as there is difficulty in communication. Nunn^5^ suggested the use of some basic actions for management. However, in most cases, we fall short of a definite plan of action for chairside behavior management of these children.

## CASE REPORT

**Fig. 1:**
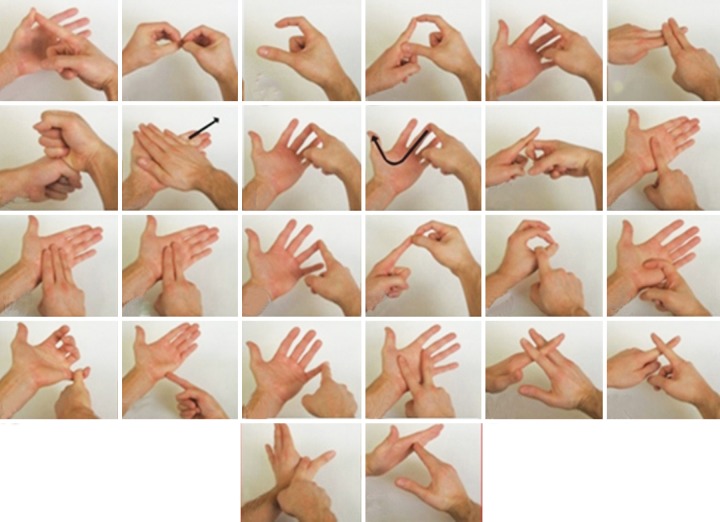
Sign language

**Fig. 2:**
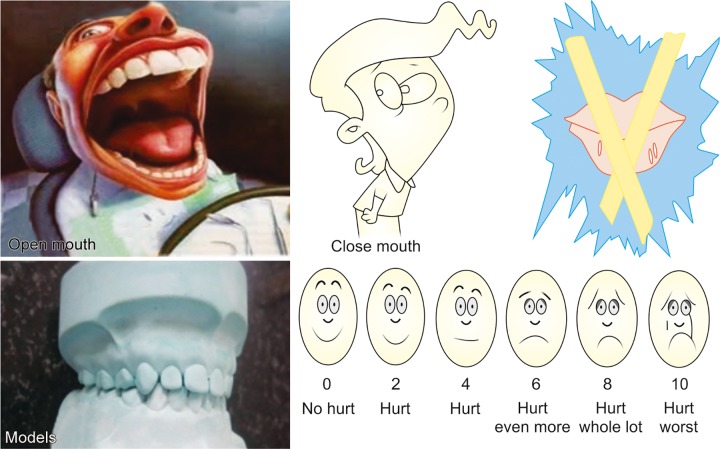
Models, pictures, rating scale

**Fig. 3:**
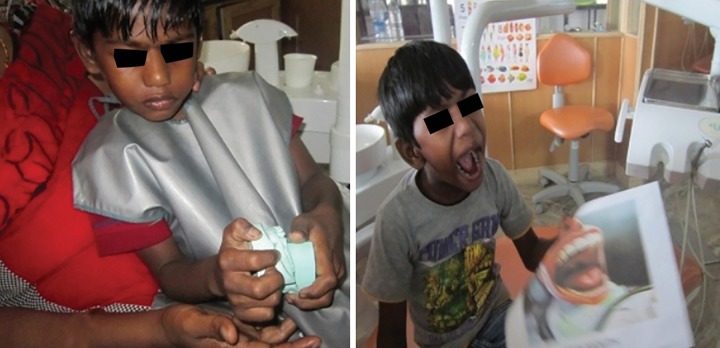
Applying second mode of management

**Fig. 4:**
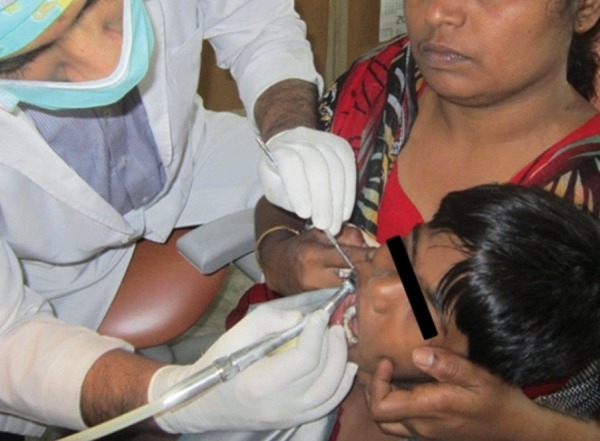
Orientation appointment

**Fig. 5:**
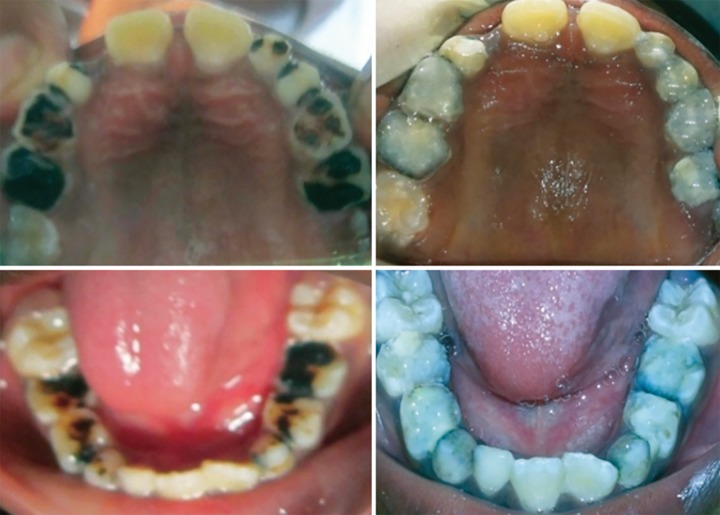
Pretreatment and posttreatment

**Fig. 6:**
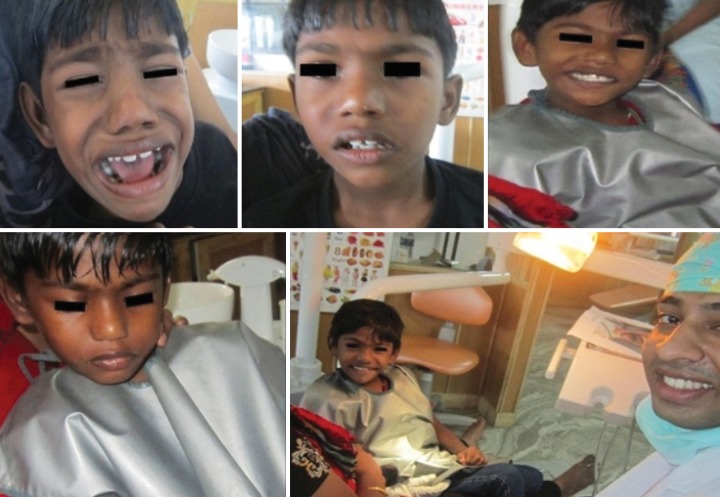
Attitude change

A 6-year-old child presented to the Pediatric Dental Clinic with caries lesions. The child was classified as having class III deafness according to the World Health Organization (WHO) classification.^6^ He was uncooperative and had a phobia toward medical practitioners, which could be attributed to prior bad experiences. Parents could not afford the child’s treatment under general anesthesia. So, chairside management was chosen, which presented a scenario encompassing all the challenges of treating a conscious special child. We devised two modes of management. The first mode was using sign language with actions (Fig. 1) and the second mode was using models, pictures, and rating scales (Fig. 2). Examination revealed arrested caries on all quadrants, so we addressed two quadrants at a time. The first method was used in the first appointment, and the second method was used in the second appointment (Fig. 3). Prior to this, an appointment was used to orient the child to both methods (Fig. 4). Restorations were successfully done (Fig. 5), and the advantages and disadvantages of both methods were unraveled. He has now developed a positive attitude toward dentistry (Fig. 6).

## DISCUSSION

**Fig. 7:**
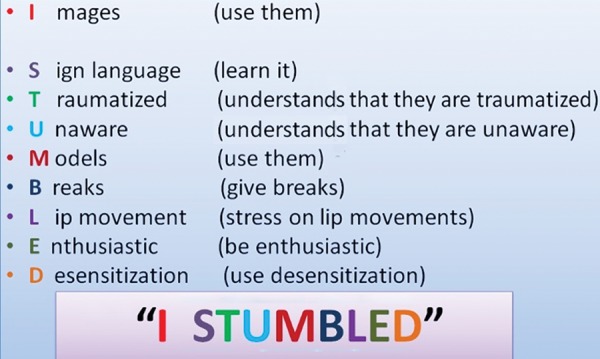
Novel strategy formulated

Hearing impaired children are facing a similar situation of not being able to undergo treatment under general anesthesia due to socioeconomic conditions and treatment at chairside being denied due to lack of awareness of management methods. Parents from low socioeconomic group do not often enroll their children in special schools at an early age, thereby affecting their communication skills.^7,8^ The sign language method was comforting for the patient, but being a child he himself was in the learning stages of sign language. The pictures and models were swift to attract the child’s attention, but distracted the child from the treatment at times. In general, the child was highly uncooperative and had increased salivation. After the management of the child who developed a positive attitude and certain key features presented themselves in managing such a child, which can be abbreviated to “I STUMBLED” (Fig. 7), the pedodontist would henceforth be prevented from stumbling while managing a deaf child. A well-constructed protocol will serve in the successful management of deaf children and is an attempt in that noble direction, which may invoke many more attempts to fabricate a concrete protocol, which will ultimately benefit deaf children.
